# Binary Theorizing Does Not Account for Action Control

**DOI:** 10.3389/fpsyg.2019.02542

**Published:** 2019-11-14

**Authors:** Bernhard Hommel

**Affiliations:** Cognitive Psychology Unit, Institute of Psychology, Leiden University, Leiden, Netherlands

**Keywords:** action control, dual-route models, goal, automaticity and control, intention

## Abstract

Everyday thinking and scientific theorizing about human action control are equally driven by the apparently obvious contrast between will and habit or, in their more modern disguise: intentional and automatic processes, and model-based and model-free action planning. And yet, no comprehensive category system to systematically tell truly willed from merely habitual actions is available. As I argue, this is because the contrast is ill-conceived, because almost every single action is both willed and habitual, intentional and automatic, and model-based and model-free, simply because will and habit (and their successors) do not refer to alternative modes or pathways of action control but rather to different phases of action planning. I further discuss three basic misconceptions about action control that binary theorizing relies on: the assumption that intentional processes compete with automatic processes (rather than the former setting the stage for the latter), the assumption that action control is targeting processes (rather than representations of action outcomes), and the assumption that people follow only one goal at a time (rather than multiple goals). I conclude that (at least the present style of) binary theorizing fails to account for action control and should thus be replaced by a more integrative view.

## Binary Theorizing on Action Control

### Will vs. Habit

The study of action control was driven by binary theorizing right from the start. In his first systematic analysis of the human will, Ach (1910) postulated that will can be best studied by analyzing the degree to, and the conditions under which, it can overcome what Ach considered its natural opponent: acquired habits. To achieve that, he developed what he called the *combined method* (“kombiniertes Verfahren”), which first established a particular habit, defined as a set of stimulus-response associations reflecting a particular stimulus-response rule, and then changed the instruction in such a way that participants were now to respond differently to the previously acquired stimulus set (see Hommel, 2000a). For instance, participants may first learn to read through lists of nonsense syllables that were followed by a rhyme (e.g., zup → tup, tel → mel) over an extended time period and then respond to the same stimulus syllables by changing the order of the letters (e.g., zup → puz, tel → let). As predicted, participants were slower and produced more errors when applying the new instruction to a stimulus set that was previously related to different responses than when working with a new set. The idea was that being exposed to lists constructed according to the first rule created stimulus-response habits that would need to be overcome in order to successfully apply the second rule. Accordingly, the degree to which participants were able to overcome the previously acquired habit (i.e., the difference in performance on old versus new sets) was taken to measure “willpower,” which was shown to differ between individuals (which was taken to diagnose individual willpower) and to vary systematically with the practice given on old sets (which was taken to increase the strength of the habit).

It is easy to see that this pioneering approach has survived until today, even though researchers less frequently take the effort to induce habits experimentally anymore: they often exploit existing habits, such as the tendency to read words even if one is to name their color, as in the notorious Stroop task (Stroop, 1935; even though Stroop himself did analyze the impact of experimental training). Like in Ach’s studies, the degree to which performance is impaired with stimulus sets that are assumed to activate the hypothetical habit (such as words denoting response-incompatible colors in the Stroop task) as compared to suitable control sets (such as nonwords, non-color words, or words denoting response-compatible colors) is taken to reflect the strength or weakness of willpower, which meanwhile has been relabeled as “cognitive control” or “executive function”—presumably in an attempt to get rid of the phenomenological connotations of the will concept (Goschke, 2003).

Working with binary oppositions such as will and habit has been taken to reflect human nature (Newell, 1973; Melnikoff and Bargh, 2018), and so it comes as no surprise that the will/habit couple has survived in various disguises until today. Its long-standing history tends to be systematically underestimated by available reviews, which for instance have dated back its introduction into theorizing about action control to the work of Tolman (1948, see Dolan and Dayan, 2013), Atkinson and Shiffrin (1968, see Monsell and Driver, 2000), or Dickinson (1985, see Gillan et al., 2015)—thus rather generously neglecting the pioneering study on the phenomenology of will by Michotte and Prüm (1911); the first systematic experimental program on studying will and habit by Ach (1910, 1935), which spanned no less than 30 years; the first approach questioning the goal-independence of habits by Lewin (1922a,b); and the other 200 or so studies on action control summarized in Ach (1935) already.

The basic thought underlying the opposition between will and habit is that some responses are so strongly associated with particular stimuli that encountering the stimulus is sufficient to activate the response. This holds for rhyming in Ach’s studies—seeing a nonsense syllables triggers the overlearned rhyming response, reading in the Stroop task—seeing the word is sufficient to trigger some reading tendency, and performing a left or right response in the Simon task (Simon, 1969)—processing a left or right stimulus triggers a spatially corresponding action (Kornblum et al., 1990). The basic setup of all tasks investigating the interplay between will and habit puts the two against each other, just as recommended by Ach (1910), by instructing individuals to carry out a relatively uncommon or counterintuitive action B to a particular stimulus that is assumed to be strongly associated with another action A. If then any experimental evidence can be found that action A was activated to at least some measurable degree, the participant is thought to have experienced an action-control problem that was due to the fact that practice established an association between A and the stimulus, so that encountering the stimulus would activate action A even under circumstances where A is not appropriate and not wanted.

Very soon after Ach’s claims that stimulus-response associations can challenge and may even outcompete the processes controlled by the actual goal, Lewin (1922a,b) reported findings calling for a more moderate view. On the one hand it was possible to counteract an intense intention with a habit that relied on few, sometimes just one repetition but, on the other, 300 repetitions were insufficient to have any impact. According to Lewin (1928), the key to understand the impact of habits has to do with their specific role in the current action plan. On the one hand, habitual actions do not represent real alternatives to intentional actions, in the sense that people would face difficulties to decide whether they should name the color of a Stroop word or read it. Lewin suggests that the intention to open a door that requires pushing the handle up, rather than down, will not be hindered by the thousands or so previous repetitions of opening doors by pushing the handle down. On the other hand, however, habitual actions do have the potency to interfere if they are embedded into a larger action context, such as if one is to open the door on one’s way to get a glass of water from the other room.

The same principle seems to apply to the Stroop effect, which is very pronounced (often >100 ms effect size) if the response set consists of spoken color words (i.e., the responses that reading the words would produce) but often dramatically shrinks or disappears with keypressing responses (e.g., McClain, 1983)—and even the effects that keypressing responses sometimes do produce seem to be artifacts due to task-irrelevant but spontaneously occurring internal naming strategies (Martin, 1978; Mascolo and Hirtle, 1990). In other words, the Stroop effect is likely to depend on introducing an obvious contradiction by requiring participants to attend to, and actually generate color words and at the same time nominally declaring color words task-irrelevant and to-be-ignored. Another obvious contradiction results from the fact that, in the standard Stroop task (as well as in other tasks following the same rationale), violating the instruction by reading the word actually pays off in 50% of the trials. This means that, on average, participants are rewarded for unintentionally or intentionally reading the word, especially given that word-reading is faster and requires less effort—just because of the more elaborate practice. That this is an important ingredient of the task is obvious from the finding that the size of the effect varies systematically with the percentage of the payoff: it becomes stronger if payoff increases and weaker if it decreases (e.g., Logan and Zbrodoff, 1979). This suggests that the impact of habitual action tendencies is anything but non-intentional, and clearly very sensitive to the expected outcome—a theme I will get back to below.

### Controlled vs. Automatic

As pointed out by Goschke (2003), theories on action control have seen a rather dramatic conceptual overhaul since the early days of Michotte, Ach, and Lewin. While the pioneering approaches were still strongly connected to the phenomenology of willing and acting, understanding which was even an explicit theoretical aim of Michotte and Ach, later theorizing preferred a less “subjective” terminology that was inspired by the increasingly popular computer metaphor for the description and analysis of human cognition in the 1950s and 1960s (Broadbent, 1958; Neisser, 1967). This terminological preference favored less colorful concepts like “controlled” versus “automatic” processing over the old-fashioned terms will and habit. Even though the basic idea was the same, the explanations changed in flavor: whereas the older approaches tried to explain the strong impact of habits by referring to an assumed cause—the strong stimulus-response association driving the habitual action, the new generation of processing theories tended to emphasize different degrees of speed and efficiency of the underlying processes (even though some studies still tested the practice-dependency of automaticity directly: e.g., Schneider et al., 1984; Smith and Lerner, 1986; MacLeod and Dunbar, 1988). For instance, the observation that responses are easier to perform in response to particular stimuli than others (e.g., left rather than write keypresses to stimuli appearing on the left) was explained by postulating the existence of a particular “population stereotype” (Fitts, 1951). At the surface, accounts of this sort do not seem to go beyond redescribing the actual finding in theoretically sounding terms, but they often implicitly rely on associationist logic: one way or another, such shared stereotypes must emerge from shared practices and training, which implies that stereotype is just another word for an associative structure linking stimuli to particular responses.

In other approaches the correspondence between controlled versus automatic processes on the one hand and will versus habit on the other is even more opaque. For instance, in their comprehensive model of stimulus-response compatibility, Kornblum et al. (1990) attribute the impact of what previously counted as habit to automaticity. It is automaticity that does the major trick in the explanation of why irrelevant stimuli seem to be able to trigger responses that conflict with the actually intended action, like in a Stroop task. Where automaticity comes from is a topic that the authors explicitly neglect: they briefly consider the possibility that training plays a role but then choose “not to make practice a major focus or concern” in their model (p. 263). Again, this renders the major theoretical contribution to the question of *why* irrelevant stimuli can trigger conflicting responses a mere reformulation of the empirical observation in theoretical terms^1^.

These and other theoretical developments indicate that the systematic replacement of the will/habit concept by the controlled/automatic concept has tempted at least more cognitively oriented theorists as cited above^2^ to refocus the theoretical attention away from the possible causes of the impact of the relevant information on action control to the consequences—away from the possible role of overlearning to the resulting automaticity. As a consequence, in these approaches automaticity was no longer defined with respect to its origin, such as the amount of training necessary to achieve it, but with respect to its opponent: the intention or control process. Note that this is a dangerous theoretical twist. The explananda targeted by control/automaticity theories derive from empirical observations that some behavior or some aspects of behavior do not fully comply with the instructions given to the investigated participants: they tend to read words rather than naming their color and press the key that spatially corresponds to the stimulus even if they should do the opposite. A certain lack of control is thus inherent in these observations, which renders the attempt to explain the observations by referring to automaticity circular: if automaticity is only defined by the absence of control, and if control is defined by compliance with the experimental instruction, the observed behavior must be automatic. In other words, automaticity cannot be an explanation because it is an integral component of the description of the to-be-explained phenomenon—automaticity is an explanandum, not an explanans!

These terminological confusions aside, it is fair to say that true automaticity has yet to be demonstrated. Kornblum et al. (1990) suggest applying the definition of Kahneman and Treisman (1984, p. 43), according to whom a strongly automatic process is one that is “neither facilitated by focusing attention on [its object] nor impaired by diverting attention from [it],” whereas “a partially automatic process is one that is normally triggered without attention directed at its object but is facilitated by having attention focused on it” (Kornblum et al., 1990, p. 261). “According to this view,” Kornblum et al. (1990) continue, “an automatic process could under some conditions be attenuated or enhanced. However, under no conditions could it be ignored or bypassed.” I have already mentioned evidence suggesting that even the Stroop effect, thought to be one of the milestones of demonstrating true automaticity, can disappear by simply changing the response set. However, such evidence might be discounted by considering a role of attention, which might be drastically reduced by this change and thus make the automaticity only partial. Moreover, Kornblum et al. claim true automaticity only for feature-overlap between stimuli and responses, which arguably is reduced, in some sense even eliminated by changing the response set in a Stroop task. However, automaticity can be shown to not exist even without changing the responses.

For instance, Valle-Inclán and Redondo (1998) presented participants with a Simon task, in which they responded to red and green colored circles by pressing the left and right response keys, respectively. In one condition, participants received the stimulus-response mapping first and were then presented with the lateralized color circle. Electrophysiological recordings showed that the presentation of the stimulus led to an increased activation in the cortical hemisphere opposite to its location—a classical lateralized readiness potential that is thought to represent response activation of the contralateral response hand (Eimer, 1995). This potential was even seen if the actual response required movement of the other hand, suggesting that it indicated the potency of the stimulus to automatically activate the spatially corresponding response hand. In another condition, the stimulus appeared first, and only thereafter the stimulus-response mapping was presented. If, according to the definition of Kahneman and Treisman and Kornblum et al., the association between stimulus location and response would be strongly automatic, the presentation of the stimulus should generate the same electrophysiological response as in the other condition. If the association would be partially automatic, the stimulus might show a reduced electrophysiological response. However, the findings showed no response whatsoever. If anything, this suggests that implementing the instruction is a precondition for automatic responses to occur, which means that they are neither fully nor partially automatic (cf., Trafimov, 2018) but what Bargh (1989) has called conditionally automatic.

A key problem with dealing with the concept of automaticity is that it remains a moving target in the literature. For instance, some authors (like Kahneman and Treisman, 1984) speak of automatic processes while others speak of automatic actions (e.g., Wheatley and Wegner, 2001). Some authors have argued that automatic processes need to meet all criteria for automaticity to deserve this label (what Moors and de Houwer, 2006, call the “all-or-none view”; e.g., Johnson and Hasher, 1987), while others were more liberal, allowing for various combinations of some of the criteria (e.g., Bargh, 1994; Moors and de Houwer, 2006), and the fact that the discussed criteria themselves vary extensively from author to author (see Melnikoff and Bargh, 2018) did not help to find a broad consensus either. For instance, while Kahneman and Treisman considered a process automatic if it is “neither facilitated by focusing attention on [its object] nor impaired by diverting attention from [it],” Bargh (1994) suggested a combination of a lack of awareness and intentionality, high efficiency, and a lack of motivation (a criterion that appeals to the desire criterion that I will criticize below), and Moors and de Houwer (2006) extend this list to eight criteria, according to which automaticity might refer to processes that are unintentional, uncontrollable, goal independent, autonomous, purely stimulus driven, unconscious, efficient, and fast.

I will not provide point-to-point point reviews of these criteria but do like to set the stage for the following discussion by means of two comments: first, the sheer number and variability of suggested criteria for sorting processes into automatic versus intentional ones, together with the fact that authors increasingly give up the idea that automaticity criteria might converge onto any coherent category (Bargh, 1994; Moors and de Houwer, 2006; Melnikoff and Bargh, 2018), undermine the original idea that cognitive processes can be categorized into two non-overlapping categories. Second, the criteria that have been suggested so far undoubtedly relate to measurable features of processes but there are reasons to doubt whether they even speak to the question of willed vs. non-willed behavior. As I will elaborate below, this is because: (1) goals and intentions control *outcomes* of behavior but not the *processes* producing it, which renders the connection between action control and criteria like controllability or autonomy questionable; (2) selecting an action emerges from the *goal-driven but fully automatic competition* between automatically executed action tendencies, which undermines the very idea that processes might be non-automatic in principle; and (3) the selection value that processes bring to this competition may well refer to the efficiency and speed of the action that this process represents, suggesting that the relevance of these criteria in action selection should be considered a sign of intentionality rather than the opposite.

### Model-Based vs. Model-Free

The most recent version of will/habit thinking comes in the disguise of models contrasting model-based and model-free systems. This contrast refers to two kinds of modeling reinforcement learning (e.g., Sutton and Barto, 2017): model-based learning is assumed to rely on a state-transition model, which accumulates knowledge about the current state, the possible actions this state allows, and the state that would follow when taking this action, and a reward model that connects end-states with particular rewards. Hence, this kind of learning is based on a kind of model of the environment, which allows forward-planning and reward-maximization even when the environment changes. Model-free learning, in contrast, does not consider sequential dependencies like state-action-outcome relationships or rewards but relies on stored selection values for all previously experienced state-action contingencies.

It is fair to say that there is no coherent theory integrating the available thoughts about how these systems work and how they interact, and it is also fair to say that quite a bit of confusion exists regarding what the terms model-based and model-free imply. One idea is that the goal-related model-based system stores contingencies between actions and outcomes while the automatic, model-free system stores stimulus-response associations (Dickinson and Balleine, 1994). According to this idea, model-based action implies consideration of the expected outcome whereas model-free action is driven by some contextual cue—a metamorphosis of the traditional habit. Others have criticized this conceptual opposition. For instance, Miller et al. (2019) have argued that the original idea assumes that habits are outcome-blind (“value-free”), whereas modern reinterpretations (e.g., Daw et al., 2005) imply that habits and model-free actions are driven by a reward-maximization process, that is, a process that depends directly on potential outcomes. Given that habit strength, the parameter that conventional habit theorists consider to be crucial for the probability to select a stimulus-response association, can well be considered a kind of selection value, the difference between value-free and value-based modeling might be less dramatic than Miller et al. (2019) assume. However, in their review on habits, Wood and Rünger (2016) question whether habits can be equated with model-free learning in view of suggestions that habits are acquired through model-based processes (Dezfouli and Balleine, 2012) and failures to find relationships between the strength of model-free learning and habit formation in individual-difference studies (Friedel et al., 2014; Gillan et al., 2015). Hence, it is clear that the model-free/model-based framework is still under development and it remains to be seen whether a systematic connection between model-based/model-free learning on the one hand and will/habit on the other will emerge. In any case, model-free action is considered to be insensitive to current action goals, whereas model-based algorithms are assumed to compute transition probabilities (e.g., an agent’s likelihood of being in a wanted state after having performed a given action), which are used to compute the expected value of actions by comparing the states they are predicted to produce to the states the agent wants to establish. Some approaches assume that the two systems compete for action control (e.g., Gillan et al., 2015), while others assume that they can be integrated (Krueger and Griffiths, 2018). Some authors consider the model-based/model-free approach a strongly advanced version of the original will/habit approach (e.g., Dolan and Dayan, 2013), while others consider the two pairs of concepts basically equivalent (e.g., Friedel et al., 2014).

However, the probably most defining two novelties in the context of the model-based/model-free approach are the contrast between action-outcome contingencies, which are related to the model-based/goal-related system, and stimulus-response associations, which are the main ingredients of the model-free/habitual system (De Wit and Dickinson, 2009), and the experimental procedure used to test whether a particular action relies on one or the other system. The latter is based on Heyes and Dickinson’s (1990) “desire criterion” of voluntary action, which together with the “belief criterion” serves as diagnostic indicator of whether a particular action is based on a goal. The belief criterion requires the voluntarily acting agent to know about the current action-outcome relation and the desire criterion requires him or her to actually want the current outcome. Given that voluntary action is commonly defined as an activity directed toward the creation of some intended effect, the belief criterion is uncontroversial and explicitly or implicitly shared by any approach to voluntary action control (see Hommel and Wiers, 2017). The role and relevance of the desire criterion is less clear, however. The key procedure to assess whether the desire criterion is fulfilled is test after satiation, which reflects the behaviorist heritage of the model-based/model-free approach and the fact that it is mainly based on experiments carried out with rodents. For instance, participants who like popcorn would be tested for popcorn-related actions before and after receiving the opportunity to eat as much popcorn as they like (e.g., Watson et al., 2014). If they would show similar attentional and behavioral biases toward popcorn after the sating procedure as they showed before the procedure, the corresponding behavioral tendency would be considered to rely on the model-free system and the stimulus-response associations it contains. The rationale for that conclusion seems straightforward: the sating procedure should make sure that participants no longer want popcorn, so if they would still be showing popcorn-approaching behavior this cannot rely on an active popcorn-getting goal—leaving a previously acquired popcorn-getting habit as the only option.

But is this rationale watertight? Let us consider why a person might eat popcorn. She may like digesting popcorn, feeling popcorn in her mouth, smelling popcorn, listening to the sound of popcorn being chewed, the attention she attracts from other popcorn-loving individuals, the satisfaction of having access to one’s favorite food, the entertainment of filling time with a liked activity, and more. Liking popcorn is thus not a simple desire for one single aspect of popcorn-eating behavior but rather a complex compound of what one might call desire aspects or subdesires. Which of those would be sated by eating as much popcorn as one likes? Being stuffed with popcorn might make the digesting aspect less attractive, but would it eliminate the joy experienced by any of the other aspects? How reasonable is it to expect that the intentional component of the behavior of a sated popcorn-lover would be identical to the behavior of a popcorn-hater or of one who just does not care about popcorn? I suggest that the fundamental flaw of satiation logic consists in the idea that agents have just one single goal and that this goal is comprehensively captured by the aspect of the goal that the sating procedure is targeting (Hommel and Wiers, 2017). While it is not impossible that this is indeed the case, it is not very likely either.

Moreover, real human actions do not only rely on more than one goal aspect, they also consist of multiple elements: eating one popcorn consists in locating it in a nearby spot, moving one’s hand toward it, opening and closing the hand until the popcorn is being grasped, moving it to one’s mouth, opening the mouth, moving the popcorn inside, dropping it, closing the mouth, and starting to chew. Most of the elements of this action pattern have been discussed as the paragon of goal-directed voluntary action in the literature on grasping (e.g., Jeannerod, 1988; Milner and Goodale, 1995), which does not seem to fit with the classification of the entire pattern as a non-intentional stimulus-driven habit. One might object that the grasping part of the action may well be intentional and the popcorn part may not, but this is exactly my point: actions commonly comprise of multiple goals and it is unlikely that any satiation procedure can ever target all of them.

Finally, if all the popcorn-related behavior of the sated popcorn-lover would really be run by the model-free system alone, why would she actually eat the popcorn? Popcorn-lovers are likely to have done many things with popcorn apart from eating: buying and putting it into the bag, carrying it home and putting it into the cupboard, unpacking it and putting it on the table, offering it to others, cleaning the table from it, and throwing the remains into the trash, and so forth. The stimulus popcorn must thus be associated with many different responses, which raises the question which of the corresponding stimulus-response habits are triggered by the popcorn after satiation. What experiments show is that even the most popcorn-loving participants show contextually appropriate behavior even after satiation: they may eat some if they stand in front of it, but they do not clean the table from it, store them, or do other things that would not fit the experimental context and the social situation it creates. If so, sating the popcorn-lover does not seem to prevent her from showing contextually and socially appropriate popcorn-related behavior, which is not well-covered by calling it model-free.

## Misconceptions in Binary Theorizing

This brief and incomplete historical tour through some of the highlights of binary theorizing on action control was intended to show that none of the suggested terminological couples really works. Practicing stimulus-response combinations is likely to change the representations thereof, and presumably makes these representations more available under certain circumstances. However, there is still no evidence that stimuli can do what intentions and goals can: to trigger a particular response. What stimuli are capable of is to trigger misleading action tendencies under circumstances that are dictated by the kind and generality of the action goal, and to the degree that they are primed and enabled by the goal, whereas the actual association strength often fails to predict the degree to which representations of stimulus-response combinations affects action control. The opposition of controlled and automatic processes suffers from similar problems and from the lack of convincing demonstrations of true automaticity. The available demonstrations are consistent with the idea that automatic processes are enabled by the goal (as suggested by Exner, 1879; James, 1890; Bargh, 1989; Gollwitzer, 1993), so that it is the goal that eventually determines whether what is considered to be an automatic process has any impact on action selection. If the model-based/model-free approach goes beyond the will/habit approach at all, which is not always clear, it does not make a convincing case that satiation procedures are a diagnostic method to tell truly goal-driven from purely stimulus-driven actions. The main problem is that this approach systematically underestimates the complexity of human action planning, a possible reflection of its behaviorist heritage. One complaint about binary theorizing has been that, even though action-control processes can be easily divided into two categories, the various categories that researchers have created so far do not sufficiently overlap to make a convincing coherent story (Melnikoff and Bargh, 2018). Even though I agree, I would even argue that the criteria offered so far have been ill-conceived and failed to allow sorting processes into non-overlapping categories. The reasons for that, I believe, have to do with some fundamental misconceptions regarding (1) the temporal relationship between the operation of processes assumed to reflect the goal and the operation of processes that are assumed to be automatic; (2) the aspects of actions that control operations keep themselves busy with; and (3) the number of goals involved in action control. In the following, I will discuss each misconception in turn.

### The Competition Misconception

When he was laying the ground for modern reaction-time-based analyses of human cognitive processes, Donders (1868/1969) was optimistic to have measured the time demands of what he called the “expression of the will.” By cleverly manipulating the cognitive demands of rather simple reaction-time experiments, and by subtracting the corresponding reaction times, Donders estimated the time demands of what we nowadays would call “response selection” in a binary-choice task to about 1/28 s. More important than the validity of this estimate is the time point at which Donders thought that the will would express itself: between processing the stimulus information and executing the response. Once we replace the outdated terms “will” and “expression of the will” through their modern successors “goal” and “controlled process,” we can see that the main function of controlled processes are thus assumed to consist in stimulus-response translation. This scenario perfectly fits with most modern action-control approaches, including the model of Kornblum et al. (1990), where the stimulus-guided “identification of the correct response” is actually the only control(led) process. It is this process that is assumed to compete with the habitual, automatic, or model-free process for controlling the eventual action.

Even though Donders’ view turned out to provide the basic theoretical template for modern action-control approaches, alternatives were available. In particular, Exner (1879) rejected the idea that the will intervenes between stimulus and response processing. Instead, he argued that preparing for a task or a particular action is accomplished by turning oneself into an automatic system long before the first stimulus appears. It is this automatic state that according to Exner enables humans to act efficiently. Note that the temporal relationship between actual control and automaticity has changed from concurrent competition to a sequence in which control operations set up the stage for automatic processes to take over. Exner’s view provides an excellent theoretical framework for understanding the observations of Valle-Inclán and Redondo (1998) discussed above: automaticity can indeed be demonstrated but it depends on the implementation of the action goal, just as the conditional-automaticity approach has claimed (Bargh, 1989). Hence, rather than competing with habitual, automatic, and model-free processes, goal-related control processes turn the cognitive system into a “prepared reflex,” as Woodworth (1938) has called it (see Hommel, 2000b).

### The Process-Control Misconception

One of the oldest theoretical problems that experimental psychology deals with relates to what Turvey (1977) has called “executive ignorance”: how is it possible that humans can carry out intentional actions but, if being asked how they did so, have very little of interest to report? The answer favored by ideomotor theorists since Lotze (1852) and James (1890) consists in the assumption of a mechanism that integrates co-activated representations of the sensory consequences of a movement (reafferent information) and the motor patterns generating these consequences. According to this view, infants and other novices start by motor babbling—performing relatively random movements—and integrate the produced motor patterns with the sensory consequences thereof (i.e., action effects). Once they have experienced action effects they like or find functional in achieving a particular goal, they “imagine,” “expect,” or “predict” these consequences, which functionally translates into reactivating the sensory representations of action effects. Given that these representations have been integrated with the motor patterns that have generated them in the past, reactivating them will prime and eventually activate the associated motor patterns, which is likely to reproduce the (now intended) sensory consequences.

Recent research has provided strong evidence for the existence of such an ideomotor mechanism, unraveled its neural and functional underpinnings, and its role in the development of intentional action (for reviews, see Hommel, 2009; Shin et al., 2010). However, for present purposes, the only important implication of this research relates to the target of control. If it is true that all that an intentionally acting agent has available are representations of past (and now expected) sensory consequences of movements, it is clear that action planning mainly consists in the activation and maintenance of these representations. In other words, action control deals with and operates on representations of expected sensory outcomes. While this might sound obvious, it is important to emphasize that this does not imply that action control is targeting particular processes. It is in fact the inability to intentionally target particular processes—the executive ignorance—that has provided the main impetus for ideomotor approaches to emerge. It thus makes little sense to compare processes that are thought to be controlled with processes that are thought to be not controlled or, as in most approaches, controlled by external stimuli. Instead, it makes more sense to assume that implementing a particular goal establishes a condition that allows representations of action-outcome relations to compete, and the representation with the closest fit to the intended action effect to win, at least under ideal circumstances (see Hommel and Wiers, 2017, for elaboration). If so, it would only be the implementation of the goal that could meaningfully be referred to as intentional or controlled, while the resulting competition would be fully automatic—just as Exner envisioned.

From this perspective, stimuli might be able to activate particular goals but, once a particular goal is implemented, they would not be able to make an agent perform an action that is entirely unrelated to that goal. And this is indeed what all available purported demonstrations of automaticity show: if a participant commits an error in a manual Stroop task, she is very unlikely to actually speak the word out loud—even though this should theoretically be the strongest habit and the most automatic tendency—but rather press the key that corresponds to the color designated by this word. Note that this error is anything but model-free, as it reflects many aspects of the task instruction, actually results from obviously outcompeting the strongest habit, and takes into account the goal of intending to press keys, rather than to say something or do something else. In other words, the error reflects the consideration of almost all aspects of the goal and the task model—something that arguably undermines all available binary accounts.

### The Single-Goal Misconception

Distinguishing between goal-related and automatic processes requires a good understanding of what the current goal actually is. Researchers implicitly or explicitly identify the current goal with reference to the instruction: aspects of the task that were considered relevant in the instruction are assumed to be represented by the goal whereas aspects of the task that were considered irrelevant are not. If thus evidence for processing the latter can be obtained, this is taken as evidence for control leakage and, thus, automatic processing. Importantly, the logic of this rationale presupposes that people have only one goal at a time, which unfortunately is entirely unrealistic. According to Atkinson and Birch (1970), the stream of human behavior is driven by multiple internal response tendencies that continuously vary in strength. Vallacher and Wegner (1987) have suggested that actions can be described at various levels, due to the concrete action plans being commonly nested into more abstract action plans, which are part of even more abstract plans, etc. Indeed, if a student is participating in a Stroop task, she is unlikely to give up her plans to earn some money, to complete her studies in time, to become a famous scientist, to be a sympathetic person, and to lead a happy life when entering the lab. How are all these goals, small-scale and large-scale, long-term and short-term, reflected in current theorizing on action control? I am afraid they are not.

That this has severe consequences for our understanding of action control can be easily shown. As discussed earlier, tasks that are thought to tap into action control give participants mixed messages about the relevance of processing particular information. In the Stroop task, words are explicitly declared to be irrelevant and yet in a substantial portion of the trials, often up to 50%, processing the word or even reading it pays off, and the argument holds for Simon tasks, flanker tasks, and many other versions of them as well. Mixed messages of this kind are likely to undermine the instructed ignorance to the type of information that the instruction has declared irrelevant. Why would a system that is assumed to be attuned to optimizing reward, as the human cognitive system, not be sensitive to the possibility to receive reward in 50% of the trials? Moreover, researchers commonly try to counteract reward-sensitive strategies by varying the irrelevant information in an unpredictable fashion. This however implies considerable variability with respect to the irrelevant stimulus dimension. Variability implies uncertainty, and the human cognitive system is notoriously interested in reducing uncertainty. This has been emphasized in recent predictive-coding approaches (Friston, 2009) but also featured strongly in the approach of Berlyne (1949, 1960). Berlyne has claimed that one of the major human drives consists in curiosity—a chronic goal that is unlikely to be traded for a Stroop instruction. Curiosity is assumed to be attracted to stimulus aspects of maximal uncertainty, which the cognitive system then tries to reduce by improving its expectations (Sokolov, 1963) or, in more fashionable terms, its predictions (Friston, 2009). If we thus assume that participants bring their curiosity goal to our labs, it should not be overly surprising that they are particularly interested in information satisfying it. If they are, this would not indicate a lack of goal-related action control but rather imply that participants satisfy various goals concurrently. Among other things, this predicts that effects hitherto assumed to reflect a leakage of control decrease as irrelevant information becomes less uncertain—which is exactly what Frings et al. (2019) have observed.

## A Unitary Alternative

As I have tried to argue, binary theorizing that divides actions into willed and un-willed categories does not provide us with a useful perspective to understand action control, neither in the disguise of the will/habit opposition, nor in the case of the intentional/automatic opposition, nor with the model-based/model-free opposition.^3^ There can be little doubt that practice changes the representation of stimulus and action events, that it creates associations between the codes forming these representations, and that these associations have impact on action control. However, there is no systematic evidence suggesting that the amount of practice can predict which actions people choose, or that people choose actions that are unrelated to their current goals. Rather, it seems that goals set the stage for the competition of various, presumably automatic processes. Given that people control goals, rather than processes, it is always possible that one of the processes being involved in the competition turns out to be less functional than others, but this is a normal outcome of processing in a system that is as competitive as the human brain. As argued and developed in some detail elsewhere (Hommel and Wiers, 2017), the time seems ripe to move on toward a more integrative framework of human action control: a framework that embraces the complexity of action control and that goes beyond mere binary categorization, both in terms of functional explanation and with respect to the neural mechanisms. In the following, I will briefly sketch the core concepts of Hommel and Wiers’ Unitary Model of Action Control (UMAC; the interested reader is referred to Hommel and Wiers, 2017, for more detail) and relate them to existing dual-route models.

According to UMAC, selecting an action is biased by multiple goals. Goals are functionally represented by one or many selection criteria that serve to provide top-down support for representations of actions that are expected to meet these criteria. For instance, the decision to grasp a cup of coffee on a table by means of one’s right hand might be driven by selection criteria that promote actions that involve grasping, actions that serve reaching a cup, actions that are likely to have positive consequences, actions that are easy to perform, and actions that go fast. The selection criteria might be taken to represent multiple goals, like quenching one’s thirst for coffee, moving with little effort, having fun, and pushing one’s energy, but UMAC does not require the specification or even the integration of dedicated goals—all that counts are activated selection criteria. Given that many of the criteria will be satisfied by more than one action representation, the (entirely automatic!) competition between suitable representations might be fierce but eventually be gravitating toward the representation of the action that best meets most or all of the criteria. Note that this scenario implies both: that all actions reflect goal states and that all actions are selected automatically. In other words, all actions are both intentional and automatic.

Highly overlearned actions or actions that the agent has preferred to choose under coffee-drinking circumstances may well have a selection benefit in the competition, because they had been learned to have low control demands (i.e., they meet the easy-to-perform criterion particularly well) and to go fast (i.e., they meet the high-speed criterion particularly well). However, it is important to emphasize that the degree of overlearning as such does not render the corresponding action special (or “more automatic”) in any way. There would be nothing wrong with calling the corresponding action a habit, simply because the agent tends to prefer this action over others—which is the defining criterion for calling something a “habit” in everyday communication. But the habitual character only exists in the eyes of the observer—the agent simply selects an action that is fast and easy. In other words, the key difference between binary theories and UMAC is that the former assume that particular actions tend to be chosen *because they are habits* that happen to be fast and efficient, whereas the latter (e.g., Moors and de Houwer, 2006) assumes that they are chosen exactly *because they are fast and efficient*. Whereas the former reasoning implies that the selection of a habit is non-intentional, at least under some circumstances, the latter implies that the selection takes place because of the current goals—which of course may involve selection criteria other than my current examples speed and efficiency.

From a UMAC perspective, it makes little sense to develop any binary system to sort actions into two categories. While practicing an action may well increase the likelihood of selecting it in the future, there is no theoretical reason to reserve a dedicated label to overlearned actions. For instance, even if overlearning to open a door by pressing the handle down, to use Lewin’s example, will make down-pressing a particularly fast and efficient action that is likely to be a strong competitor for selection under high-speed pressure (a selection criterion that propagates fast and efficient actions), a strong accuracy instruction is likely to render this candidate entirely impotent. Note that this theoretical problem cannot be solved by turning the binary distinction between intentional action and habit into a continuous dimension; it rather highlights the actual status of the word “habit,” which should be considered a descriptive term taken from everyday language but not a scientific, and certainly not an explanatory concept (cf., Hommel, in press).

An obvious objection against UMAC might be that it is merely changing the semantics in a way that is impossible to test: every time some seemingly non-intentional behavior can be demonstrated, a new goal might be invented to account for it. This would indeed not do a good service to our understanding of action control, but fortunately UMAC is not at all immune to empirical test, as the following examples show. First, a key point of UMAC is that implementing an action goal/intention enables (increases the possible impact of) event representations with features from dimensions that either are or seem to be task-relevant. It is this task-relevance that renders the tendency to say “red” in a Stroop task a potent competitor in action selection. A strict automaticity approach could thus easily disconfirm the corresponding UMAC prediction by demonstrating that people say “red” when being faced with the word “RED” in the absence of any task or in a task that neither requires reading nor otherwise dealing with colors or color words. Second, even though it may be difficult to create conditions under which chronic goals like curiosity or novelty-seeking can be entirely switched off, it is certainly possible to create conditions that make that goal more or less relevant, like in a dual-task paradigm with one task emphasizing or not emphasizing novel information. Demonstrating that such a manipulation has no impact on the processing of novel information whatsoever would be difficult to take for UMAC.

Another interesting issue in the comparison of UMAC and strict automaticity approaches relates to the role of external stimuli. Both approaches assume that action alternatives can be activated by processing external stimuli: the automaticity approach assumes that processes and even actions can be triggered by stimuli—where the latter, as I have argued above, is yet to be demonstrated in humans—and UMAC assumes that stimuli activate all representations that feature-overlap with the stimulus on task-relevant dimensions (Hommel, 2004; Hommel and Wiers, 2017). The critical difference between these two theoretical approaches does thus not relate to the possibility of stimulus-induced activation of internal representations but rather to the question whether the degree of this activation is moderated by task-relevance (which UMAC assumes but the automaticity approach does not) and whether activation can result in action, as the automaticity approach assumes, or in competition for action control according to goal criteria, as UMAC suggests.

Yet another difference refers to the role of the context. Many automaticity accounts imply a rather pure, de-contextualized connection between particular stimuli and overlearned responses to these stimuli (e.g., Dickinson, 1985; De Wit and Dickinson, 2009). In contrast, UMAC assumes that the basic representational unit is an event file (Hommel, 2004), which integrates stimuli, actions, and outcomes, as well as internal and external context conditions. This feature allows UMAC to deal with findings as those reported by Neal et al. (2011). These authors found that participants who are used to eating popcorn in the cinema are likely to eat popcorn even if it is stale and even though they report disliking it, but only if it is offered in the cinema but not in a lab room while watching music videos. Even though more research is required to identify further conditions of such observations, UMAC’s assumption that action representations are contextualized and, thus, more likely retrieved and more strongly activated in a context in which they were acquired, is well-equipped to tackle such empirical challenges in principle.

Last but not least, it is important to point out that UMAC does not deny the important role of practice—the key player of automaticity accounts. According to UMAC, practice can change behavior in various ways that have an impact on action control, that is, on the probability that the event file related to a practiced action is eventually selected for execution. For instance, practice is known to increase the speed and efficiency at which an action is carried out. Increasing practice will thus increase the number of event files that satisfy goals that emphasize or imply speed and efficiency, which will make these event files more likely to outcompete others if and to the degree that these goals are activated. Practice will also lead to a more systematic, sharpened integration of other action effects, so that the experienced popcorn-eater, say, will have learned and will thus anticipate a richer and more specific set of sensory outcomes of popcorn eating than the popcorn greenhorn. This in turn will make the resulting event files more potent competitors under conditions in which goals that are satisfied by such outcomes are activated. For instance, it may take some time to register and appreciate social-improvement signals from other popcorn-eaters in the cinema, so that popcorn eating is more likely to satisfy social goals in the more experienced popcorn-eater. Practice may also increase or reduce the role of context, depending on the kind of experience: if 90% of the event files resulting from one’s street-crossing experience contain a representation of a green light, encountering a green light is likely to play a stronger role in selecting the appropriate action than if light representations in street-crossing event files would be much more varied. UMAC and automaticity accounts do thus not differ with respect to the assumption that practice and learning can have a strong impact on action control, but they rather differ with respect to why and how this impact is thought to be achieved. If, thus, the popcorn-lover keeps eating popcorn even after having finished an XXL tube, this might reflect the ongoing satisfaction of (e.g., tactile, olfactory, or social) goals that are not yet sated, or simply an attempt to fight boredom, rather than a breakdown of intentionality.

## Conclusion

The unitary account to action control shows that there is no need to heed the conventional distinction between will and habit. In this framework, goals still play an important role, as do automatic processes and practice, but goals and automatic processes do not compete but serve complementary purposes. The next challenge will be to better understand how goals and selection criteria constrain the operation of automatic processes, and when and under which circumstances action representations become relevant competitors in the action-selection process. In any case, I believe that theorizing about action control is ready to take the next step, and that this next step should not consist in inventing yet another binary opposition.

## Author Contributions

The author confirms being the sole contributor of this work and has approved it for publication.

### Conflict of Interest

The author declares that the research was conducted in the absence of any commercial or financial relationships that could be construed as a potential conflict of interest.

## Footnotes

^1^According to Lewin (1931), the idea that categorizing a particular phenomenon is sufficient to explain it is a reflection of what he called Aristotelian psychology (a theoretical attitude that is very typical for stage approaches to studying human information processing: Hommel, in press), which he contrasts with Galilean psychology that seeks to unravel the actual functional mechanisms.

^2^This is not to say that attempts to systematically control the degree of automaticity acquired through experimental practice no longer exist. The learning-theoretical tradition to make training/exercise part of the experimental design has survived especially in the cognitive neurosciences (e.g., Schwabe and Wolf, 2009) and applied areas related to lifestyle issues and addiction (e.g., Watson et al., 2014;  Lin et al., 2016).

^3^Note that what I criticize is the way theorists have sorted actions, action-control operations, and related processes into two categories over the last 150 years or so. It is thus *a particular kind of binary theorizing* that I criticize, and my main argument is that the distinctions being drawn between the binary categories make little sense both theoretically and empirically. I would like to emphasize that I am more interested in the flaws in making these distinctions than in the binary nature of the underlying theorizing. Accordingly, theories that would keep that distinction but add further categories would not escape my criticism. Conversely, binary theories that make other distinctions than between willed and un-willed (and related versions) may well escape it, even though I find it difficult to imagine what kind of distinction that might be and even though I would suspect that it would still tempt researchers to categorize actions and related processes rather than understanding their mechanics (the tendency that I criticize in Hommel, in press).
